# NR3C1 is required for normal somatotrope differentiation and *Foxo1* expression in pituitary

**DOI:** 10.1210/endocr/bqag060

**Published:** 2026-05-16

**Authors:** Pratyusa Das, Ridwanullah A Abubakar, Nooshin Mojahed, Dania Abou-Jabal, Prisha Kittu, Michelle L Brinkmeier, Jessica Olsen, Dale Ann Sunny, Sally A Camper, Robin Lovell-Badge, Karine Rizzoti, Buffy S Ellsworth

**Affiliations:** Division of Molecular and Integrative Physiology, Southern Illinois University School of Medicine, Carbondale, IL 62901-6523, USA; Division of Molecular and Integrative Physiology, Southern Illinois University School of Medicine, Carbondale, IL 62901-6523, USA; Division of Molecular and Integrative Physiology, Southern Illinois University School of Medicine, Carbondale, IL 62901-6523, USA; Division of Molecular and Integrative Physiology, Southern Illinois University School of Medicine, Carbondale, IL 62901-6523, USA; Division of Molecular and Integrative Physiology, Southern Illinois University School of Medicine, Carbondale, IL 62901-6523, USA; Department of Human Genetics, University of Michigan Medical School, Ann Arbor, MI 48109, USA; Genetic Modification Service, The Francis Crick Institute, London NW1 1AT, UK; Division of Molecular and Integrative Physiology, Southern Illinois University School of Medicine, Carbondale, IL 62901-6523, USA; Department of Human Genetics, University of Michigan Medical School, Ann Arbor, MI 48109, USA; Laboratory of Stem Cell Biology and Developmental Genetics, Francis Crick Institute, London NW1 1AT, UK; Laboratory of Stem Cell Biology and Developmental Genetics, Francis Crick Institute, London NW1 1AT, UK; Division of Molecular and Integrative Physiology, Southern Illinois University School of Medicine, Carbondale, IL 62901-6523, USA

**Keywords:** glucocorticoid, NR3C1, FOXO1, somatotrope, lactotrope, *Prop1-cre*

## Abstract

Glucocorticoids are an important signal for the differentiation of many types of cells. Consistent with this, several studies have demonstrated that glucocorticoids promote the somatotrope differentiation program and functionality. Interestingly, we previously found that loss of the forkhead factor, FOXO1, results in delayed emergence of somatotropes and prevents glucocorticoid-induced premature differentiation of somatotropes. In the present study, we find that pituitary-specific deletion of *Nr3c1*, the gene encoding the glucocorticoid receptor, impairs somatotrope differentiation and increases lactotrope numbers embryonically and at 5 days after birth. The number of somatotropes remains reduced at age 7 weeks in females and males, but lactotrope cell numbers are increased only in females at this age. FOXO1 is nearly undetectable in pituitary glands from mouse embryos lacking NR3C1 and continues to be reduced in adults. These findings suggest that glucocorticoids are an important signal for determining the balance between somatotropes and lactotropes and that FOXO1 may mediate NR3C1 induction of somatotrope differentiation.

The pituitary gland plays a pivotal role in regulating a wide array of physiological processes and is divided into 3 distinct sections in rodents: the anterior, intermediate, and posterior lobes. In all vertebrates the anterior pituitary is composed of 5 specialized cell types, each responsible for producing specific hormones that target different organs in the body. Lactotropes secrete prolactin (PRL), somatotropes produce growth hormone (GH), gonadotropes are responsible for the secretion of the gonadotropins, follicle-stimulating hormone and luteinizing hormone, corticotropes produce adrenocorticotropin (ACTH), and thyrotropes produce thyrotropin (TSH). The intermediate lobe contains melanotropes, which secrete melanocyte-stimulating hormone. Lastly, the posterior pituitary is of neuronal origin and stores and releases antidiuretic hormone and oxytocin (1-3).

Despite variations in the detailed morphology and organization of the pituitary gland across different species, the general principles governing its development, structure, and the molecular pathways involved in the differentiation of its cell types are conserved across vertebrates. For this reason, studies in many species have contributed to our understanding of how this small gland develops (2). In mice, the earliest sign of pituitary development is evident around embryonic day (e)8.5, with the formation of the hypophyseal placode (1, 4). Around e9, this placode begins to form the rudimentary Rathke pouch (RP), which eventually gives rise to the anterior and intermediate lobes of the pituitary gland. By e10.5, a specific region of the ventral diencephalon, located above the RP, starts to develop into the infundibulum. This region will give rise to the posterior pituitary and the stalk that connects the pituitary to the hypothalamus. The close physical relationship between the RP and the ventral diencephalon is maintained throughout the early stages of pituitary development (1, 2). Patterning and cell specification of the anterior pituitary gland is dependent on signals from this region, referred to as the pituitary organizing center, such as BMP, FGF, SHH, and WNT (1, 2, 5, 6). These factors are important for activating many transcription factors that regulate pituitary gland development. For example, the WNT pathway is involved in activation of *Pitx2* (7) and β-catenin can interact with PROP1 (1, 8).

Pituitary cell differentiation occurs through several steps (1, 2, 9). In the first step, lineage specific transcription factors are activated. POU1F1 promotes commitment of the POU1F1-lineage which will differentiate into somatotropes, lactotropes, or thyrotropes (10-12). TBX19 is expressed in the POMC lineage serving as the progenitor for corticotropes and melanotropes (13, 14). Finally, NR5A1 is present in the progenitor cells that will become gonadotropes (15, 16). In the second step, these lineages further differentiate into subpopulations to form specific cell types. For example, PAX7 specifies the melanotrope fate through its pioneer activity, shifting the epigenetic landscape to allow TBX19 to activate melanotrope-specific genes (17, 18). The signals that are responsible for divergence of somatotropes and lactotropes are less well understood. A number of factors are enriched in lactotropes (NR4A1, NR4A2, POU4F1) and somatotropes (NR3C2, RXRG, NUPR1) (19) and several factors demonstrate roles in somatotrope differentiation (NEUROD4, FOXO1) (20-23). During the third step, lineages expand in response to specific signals. For example, GH-releasing hormone (GHRH) stimulates the expansion of somatotropes (24-26) and thyrotropin-releasing hormone leads to expansion of the thyrotrope population (27). In the final phase, these highly specialized, committed cells increase in size and translational capacity to allow for their production of high levels of hormones (28, 29).

Glucocorticoid signaling has been implicated in somatotrope differentiation (30-34). In the present study, we provide evidence that glucocorticoids act at key steps in this process. We show that loss of the glucocorticoid receptor, NR3C1, causes a shift in the divergence between somatotrope and lactotrope cells, suggesting that glucocorticoid signaling is key for refining cell commitment between somatotropes and lactotropes. Additionally, loss of NR3C1 impairs expansion of the somatotrope lineage postnatally. Thus, glucocorticoid signaling is critical for multiple steps in somatotrope differentiation.

We have previously shown that glucocorticoids increase expression of the forkhead transcription factor, *Foxo1*, and that loss of *Foxo1* in the pituitary gland leads to a delay in somatotrope differentiation and reduced somatotrope function (20, 21, 35). In addition, we found that loss of *Foxo1* impaired glucocorticoid-induced somatotrope differentiation (21). In the present study we observe a drastic reduction in the number of pituitary cells expressing FOXO1 when *Nr3c1* has been deleted, suggesting that FOXO1 may, in part, mediate the effects of glucocorticoids during somatotrope differentiation.

## Materials and methods

### Mice

All animal experiments performed at the Francis Crick Institute were approved under the UK Animals (Scientific Procedures) Act 1986 and under the project license PP8826055 and PP5939090 and by the Francis Crick Animal Welfare and Ethical Review Body (AWERB). Adult mice used in this study were age 7 weeks. The e18.5 time points were generated at Southern Illinois University, and these experiments were approved by the Southern Illinois University Animal Care and Use Committee in accordance with the National Institutes of Health Guide for the Care and Use of Laboratory Animals.

*Prop1^T2AiCre^* mice were generated by the Genetic Modification Service at the Francis Crick Institute using CRISPR-Cas9–assisted targeting to insert an improved Cre recombinase into the 3′ end of the gene replacing the stop codon of exon 3, via a GSG and T2A peptide linker and followed by a stop codon. The donor template contained a 1158 bp insert of GSG, T2A, and iCre, with 681 bp of homology at the 5′ end and 825 bp of homology at the 5′ end. The AAV donor vector was synthesized and packaged into AAV serotype 1 by VectorBuilder. The guide sequence was 5′-CCTGGAACTAAGTGGTGATA-3′ (Table 1) and was synthesized as a synthetic guide RNA by Integrated DNA Technologies. CRISPR Cas9 reagents were transduced by AAV into 1-cell C57BL/6J zygotes as previously described (36).

**Table 1:** Oligonucleotide sequences

Name	Sequence (5′-3′)
gRNA	cctggaactaagtggtgata
iCre F	tcctgggcattgcctacaac
iCre R	cttcactctgattctggcaatttcg
iCre Probe	accctgctgcgcattg
Prop1-2 WT F	catgtttcccctcagccttga
Prop1-2 WT R	gttatggagagagggtgccttatc
Prop1-2 WT Probe	aaagtcctggaactaagtgg
LR-5F	gatgccctaaggttctcggg (from sequence upstream of 5′ homology arm)
LR-5R	ggttttggtgcacagtcagc (within iCre sequence)
LR-3F	ccaaggatgactctgggcag (within iCre sequence)
LR-3R	gcacacacacacacacagaa (from seq downstream of 3′ homology arm)
iCre CNV qPCR F	tccctggtgatgaggagaat
iCre CNV qPCR R	tctccatcagggatctgactt
iCre CNV qPCR Probe	tggcctttgaacgcactgactttg
LOA qPCR F	ccctcagccttgagacg
LOA qPCR R	gtttcaagagggttatggagagag
LOA qPCR Probe	cctggaactaagtggtgataaggcacc
ONT-5F	ggacagcaggctagaccatc (from sequence upstream of 5′ homology arm)
ONT-5R	gggacacagcattggagtca (within iCre sequence)
ONT-3F	ggtgcaagctgaacaacagg (within iCre sequence)
ONT-3R	agcaacaggatggacacagg (from seq downstream of 3′ homology arm)

Abbreviations: gRNA, guide RNA; qPCR, quantitative polymerase chain reaction; WT, wild-type.

Transnetyx quantitative polymerase chain reaction (qPCR) was performed on mouse biopsies to identify 4 F0 mice that were preliminarily positive for insertion of the donor template. qPCR was performed for copy number assessment (see Table 1, iCre qPCR). 5′ and 3′ Long-range PCR covering the entire insert (see Table 1, LR primers) was also performed and Sanger sequencing performed for 3 F0 mice. Three founder mice were identified and crossed with C57BL/6J mice, of which only one showed germline transmission of the insert. Transnetyx qPCR identified 9 putative F1 heterozygous mice from one founder, which were further validated as described for the F0 mice. Long read sequencing (Oxford Nanopore Technologies) was performed on overlapping 3- to 4-kb amplicons spanning the targeted insertion. Characterization of *Prop1^+/T2AiCre^* mice was performed by mating them to tdTomato reporter mice (Jackson Laboratories, strain No. 007909) (37).

*Nr3c1^fl/fl^* in which *loxP* sites flank exon 3 of the *Nr3c1* gene (*tm1Gsc*, MGI:1931329) (38) were crossed with *Prop1^+/T2AiCre^* mice to ultimately obtain *Nr3c1^fl/fl^;Prop1^+/T2AiCre^* mice. For studies at e18.5, *Nr3c1^fl/fl^* in which *loxP* sites flank exon 3 of the *Nr3c1* gene (*tm1.1Jda*, MGI:5447468, Jackson Laboratory, strain No. 021021) (39) were crossed with *Prop1^+/T2AiCre^* mice. Mice were maintained on a C56BL/6J background and were housed in isolators with a light cycle of 12 hours day and 12 hours night with a dawn to dusk setting, at a temperature of 22 °C ± 2 °C with humidity of 55% RH ± 10%. The day the copulatory plug was observed is considered e0.5.

### Immunohistochemistry

*Nr3c1^fl/fl^;Prop1^+/T2AiCre^* mutants were compared to *Nr3c1^fl/fl^ Nr3c1^+/fl^,* and *Nr3c1^fl/+^;Prop1^+/T2AiCre^* controls. Tissues were fixed in 4% formaldehyde in phosphate-buffered saline overnight at 4 °C and dehydrated in a graded ethanol series before paraffin processing. Sections of 5 μm were deparaffinized in xylene and rehydrated in graded ethanol. Antigen retrieval was performed by boiling slides in 10 mM citric acid buffer, pH 6.0, for 5 to 10 minutes according to the protein being detected. All primary antibodies were diluted as detailed in Table 2 and incubated on tissue sections overnight at 4 °C in a humidified chamber. For detection of GH (catalog No. AFP5672099, RRID: AB_2721132 [http://antibodyregistry.org/AB_2721132]) and NR3C1 (catalog No. 12041, RRID: AB_2631286 [https://scicrunch.org/resolver/RRID: AB_2631286]), following primary antibody incubation, sections were incubated with a biotinylated secondary antibody for 60 minutes followed by streptavidin (SA)-conjugated fluorescein isothiocyanate or SA-conjugated tetramethylrhodamine isothiocyanate (TRITC) incubated at room temperature for 30 minutes. For PRL (catalog No. AFP107124012, RRID: AB_3683723 [https://scicrunch.org/resolver/RRID: AB_3683723]) detection, following incubation with primary antibody as described earlier, sections were incubated with TRITC-conjugated secondary antibody for 1 hour at room temperature. For detection of FOXO1 (catalog No. 2880, RRID: AB_2106495 [https://scicrunch.org/resolver/RRID: AB_2106495]) and POU1F1 (catalog No. ab10623, RRID: AB_297352 [https://scicrunch.org/resolver/RRID: AB_297352]), immunohistochemistry (IHC) was performed using the CF Dye 543 Tyramide kit (Biotium, catalog No. 92172) or the CF Dye 488A Tyramide kit (Biotium, catalog No. 33002), depending on the color desired, as per the manufacturer's directions. Specifically, following incubation with primary antibody as described earlier, slides were incubated with a biotinylated secondary antibody at room temperature for 60 minutes followed by incubation with SA-horseradish peroxidase for an hour and the appropriate fluorescent tyramide label (CF543 or CF488) for 10 minutes. Before mounting coverslips, nuclei were stained with 4′,6-diamidino-2 phenylindole (DAPI) for 5 minutes at room temperature.

**Table 2:** Antibody information

Name	ID	Antigen	Provider	Cat. No.	Dilution
Rabbit anti-mouse GH antibody	AB_2721132	GH1	NHPP	AFP5672099	1:100
Anti–growth hormone antibody	AB_11128284	GH1	Abcam	ab126882	1:500
FOXO1 (C29H4) rabbit mAb	AB_2106495	FOXO1	Cell signaling technology	2880	1:50
NR3C1 (D6H2L) rabbit mAb	AB_2631286	NR3C1	Cell signaling technology	12041	1:100
Rabbit anti-human GHRHR	AB_1566283	GHRHR	Abcam	ab76263	1:400
Anti-Pit1 [2C11]	AB_297352	POU1F1	Abcam	ab10623	1:100
Rabbit anti-recombinant PRL	AB_3683723	PRL	NHPP	AFP107124012	1:50
ACTH	AB_2313902	POMC	NHPP	AFP156102789	1:100
Rat anti-mouse TSHB	AB_2923323	TSHB	NHPP	AFP1274789	1:200
Guinea pig anti-rat LHB	AB_2665565	LHB	NHPP	AFP22238790GPOLHB	1:500
Goat anti-SOX2	AB_355110	SOX2	Biotechne	AF2018	1:500
Anti-human FOXL2	AB_3730166	FOXL2	Reiner A. Veitia	ALT_C-hFOXL2	1:500

Abbreviations: ACTH, adrenocorticotropin; GH, growth hormone; PRL, prolactin.

In double IHC, for NR3C1/GH and FOXO1/NR3C1, sections were stained using the same reagents described earlier. Following the immunostaining protocol for the first antigen, but before staining with DAPI, sections were incubated with FAB block (Jackson Immuno Research, catalog No. 124362) for 30 minutes at room temperature. Then staining for the second antigen was performed, as described earlier. Before mounting coverslips, nuclei were stained with DAPI for 5 minutes.

IHC images were taken using a Retiga 2000R digital camera attached to a Leica DM 5000B fluorescent microscope. Individual pictures of fluorescent channels were merged using Image J. Some images were brightened for illustrative purposes; however, alterations were replicated both in control and experimental images to maintain the ability to compare results.

For quantification of immunopositive cells at P5 and age 7 weeks, the anterior lobe of pituitary glands was dissociated, plated on slides, and fixed as described (40). Immunofluorescent staining was then performed to detect anterior pituitary hormones luteinizing hormone (catalog No. AFP22238790GPOLHB, RRID: AB_2665565 [https://scicrunch.org/resolver/RRID: AB_2665565]), TSH (catalog No. AFP1274789, RRID: AB_2923323 [https://scicrunch.org/resolver/RRID: AB_2923323]), GH (catalog No. ab126882, RRID: AB_11128284 [https://scicrunch.org/resolver/RRID: AB_11128284]), POMC (catalog No. AFP156102789, RRID: AB_2313902 [https://scicrunch.org/resolver/RRID: AB_2313902]), and PRL (catalog No. AFP107124012, RRID: AB_3683723 [https://scicrunch.org/resolver/RRID: AB_3683723]) (see Table 2). Secondary antibodies that are directly conjugated to fluorophore were used to label the antigen. Automated counting was performed after scanning of the slides using QuPath-0.3.2 (https://qupath.github.io/), as described previously (40). *Nr3c1^fl/fl^;Prop1^+/T2AiCre^* mutants were compared to *Nr3c1^fl/fl^, Nr3c1^fl/+^, Prop1^+/T2AiCre^* controls.

To ensure specificity of primary antibodies in IHC studies, a no primary control was used. When available, a tissue section lacking the antigen was also used as a negative control.

### RNAscope in situ hybridization

*Nr3c1^fl/fl^;Prop1^+/T2AiCre^* mutants were compared to *Nr3c1^fl/fl^ Nr3c1^+/fl^,* and *Nr3c1^fl/+^;Prop1^+/T2AiCre^* controls. Embryo heads were fixed in 10% formalin overnight at 4 °C and dehydrated in a series of increasing concentrations of ethanol. Tissues were embedded in paraffin, cut into 5-μm sections, and mounted on SuperFrost Plus slides (Fisher Scientific, catalog No. 1255015). In situ hybridization was performed using the RNAscope 2.5 HD Reagent Kit-RED (ACD Bio, catalog No. 322360) for single-probe detection and mouse *Ghrh* probe (ACD Bio, catalog No. 470991). Sections were baked at 60 °C for 1 hour. Sections were deparaffinized in xylene, rinsed twice in 100% ethanol, and air-dried for 5 minutes at room temperature. Sections were incubated with RNAscope Hydrogen Peroxide (ACD Bio, catalog No. 322335) for 10 minutes at room temperature and rinsed twice in RNase-free water. For target retrieval, sections were acclimated in RNase-free water for 10 seconds before being placed in a steamer containing 1× Target Retrieval Reagent (ACD Bio, catalog No. 322000) at 100 °C for 15 minutes. Sections were rinsed in RNase-free water, incubated in 100% ethanol for 3 minutes, and dried at 60 °C for 5 minutes. Sections were treated with Protease Plus (ACD Bio, catalog No. 322331) for 30 minutes at 40 °C, then rinsed twice in RNase-free water. Probes were hybridized to sections at 40 °C for 2 hours in a humidified chamber. Sections were washed twice in 1× wash buffer for 2 minutes with agitation at room temperature. Sections were incubated sequentially with AMP 1 to 5 for 30 minutes each at 40 °C, washing with 1× wash butter for 2 minutes at room temperature with agitation after each incubation. Sections were incubated with AMP 6 for 15 minutes at 40 °C then washed in 1× wash buffer for 2 minutes at room temperature with agitation. Sections were incubated in a 1:60 ratio of Fast RED-B to Fast RED-A for 10 minutes at room temperature followed by 2 rinses in tap water. Sections were counterstained in 50% Harris hematoxylin (41) and washed in tap water 3 to 5 times with agitation until slides were clear, while sections remained purple. Sections were briefly rinsed in 0.02% ammonium hydroxide 3 to 5 times with agitation until they turned blue. Slides were baked at 60 °C for 15 minutes, or until completely dry. Sections were dipped in xylene and immediately mounted in EcoMount (ACD Bio, catalog No. 320409).

### Data and statistical analysis

Data from single-cell RNA sequencing (scRNAseq) of adult male pituitary glands (42) was reanalyzed using Seurat (43). Count data from immunohistochemical studies for individual animals are represented with a minimum number of 3 animals/age/sex. We counted a minimum of 5000 cells/animal. Because somatotropes represent the largest (in male) and second largest (in female) proportion of pituitary endocrine cells, their loss in *Nr3c1^fl/fl^;Prop1^+/T2AiCre^* mutants lead to an artifactual increase in the proportion of all other endocrine cell types. To avoid this bias, we corrected the total number of cells in mutants by adding the missing cells (we extrapolated the average cell count from age- and sex-matched controls). We then used a beta regression model to assess the statistical significance of endocrine cell proportion differences between mutants and controls. Statistical analyses were performed using R studio.

## Results

### NR3C1 is expressed in the majority of somatotropes and corticotropes

**Figure 1:**
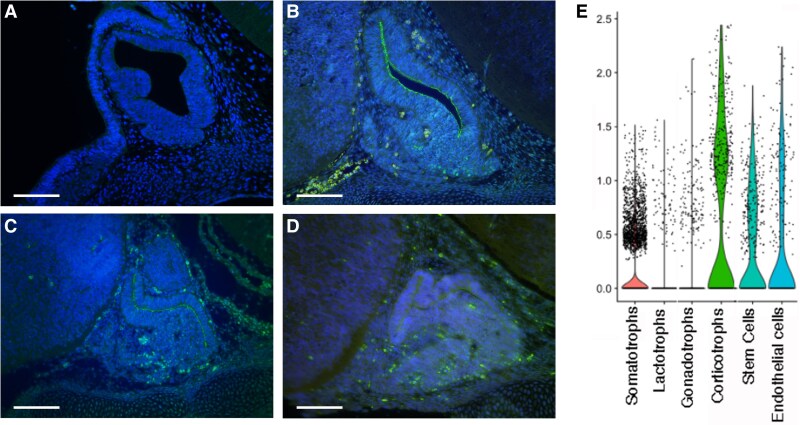
NR3C1 protein is localized to the nucleus of pituitary cells throughout development. Immunohistochemistry for NR3C1 on midsagittal pituitary sections at A, e12.5; B, e13.5; C, e14.5; and D, e15.5. Nuclei are stained with DAPI (4′,6-diamidino-2-phenylindole). Scale bars represent 100 μm. The experiments were replicated independently in 3 different litters. E, Violin plots of single cell RNA sequencing data from 7-week-old male mice (42) show that *Nr3c1* is expressed in all pituitary cell types and is enriched in somatotropes, corticotropes, and stem cells.

Glucocorticoids, such as cortisol and corticosterone, signal primarily through the glucocorticoid receptor, NR3C1. *Nr3c1* transcripts are detectable in the pituitary gland as early as e10, with a statistically significant increase by e12, as demonstrated by Northern blot analysis. However, *Nr3c1* expression was not detectable until e12 via in situ hybridization (44, 45). To evaluate the spatial and temporal expression patterns of NR3C1 at the protein level, we performed IHC on sections of mouse embryonic pituitary. Our data reveal that NR3C1 protein is apparent in the pituitary gland by e13.5. Following this initial appearance, NR3C1 levels progressively increase in the pituitary gland throughout development, with its localization predominantly in the nucleus, reflecting its function as a transcriptional regulator (Fig. 1). Analysis of scRNAseq data from adult mice (42) demonstrates that *Nr3c1* is widely expressed in the adult mouse pituitary gland (Fig. 1E).

**Figure 2:**
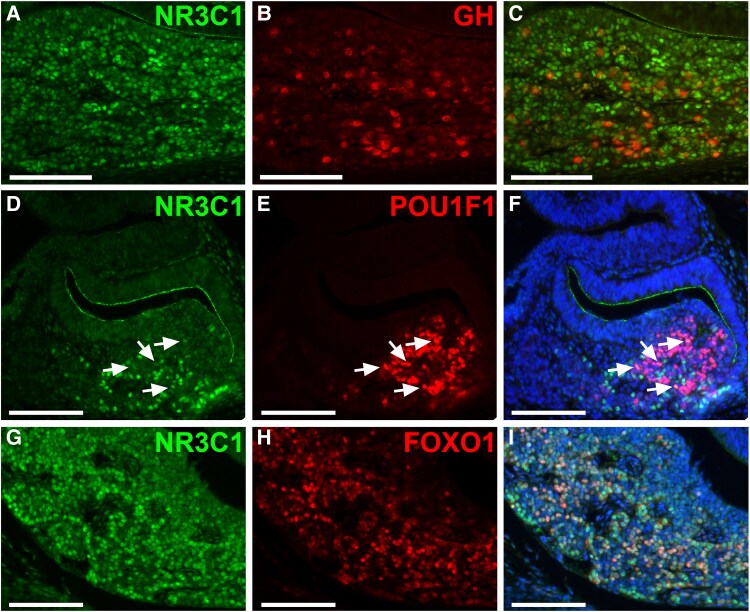
The majority of somatotropes contain NR3C1 in the pituitary. A to C, Immunohistochemistry for NR3C1 (A) and growth hormone (GH) (B) in midcoronal sections of the pituitary at e16.5 show many cells with nuclear NR3C1 and cytoplasmic GH (C, merged image). D to F, POU1F1 (E) colocalizes with a few NR3C1-positive (D) cells, as indicated by arrows, in midsagittal sections at e14.5, (F, merged image). G to I, Nuclear NR3C1 (G) is present in most FOXO1-positive cells (H) in midcoronal sections at e16.5, (I, merged image). F and I, Nuclei are stained with DAPI (4′,6-diamidino-2-phenylindole). Scale bars represent 100 μm. The experiments were replicated independently in 3 different litters.

NR3C1 has been localized to somatotrope cells in chicken embryos (32), adult rats (46). We find that NR3C1 is present in nearly all GH-positive cells in mouse embryos at e16.5, colocalizing with GH in somatotropes (Fig. 2A-2C). While GH was confined to the cytoplasm, NR3C1 was predominantly localized in the nucleus. Somatotrope cells differentiate from POU1F1-positive progenitor cells (10, 11). Consistent with a role for NR3C1 in promoting somatotrope differentiation, a subset POU1F1-positive cells contain NR3C1 (Fig. 2D-2F). FOXO1 also plays a critical role in the development of mouse somatotropes (20). Some evidence suggests that transcription of *Foxo1* may be regulated by glucocorticoid signaling. For example, in skeletal muscle cells, glucocorticoids upregulate *Foxo1* expression through glucocorticoid response elements, contributing to muscle atrophy (47). We find that glucocorticoids stimulate *Foxo1* expression and increase FOXO1 nuclear localization in the pituitary glands of mouse embryos (30). To determine if FOXO1 is present in NR3C1-positive cells in the pituitary gland, our findings show that most FOXO1-positive cells colocalize with NR3C1 at e16.5, with NR3C1 predominantly localized in the nucleus (Fig. 2G and 2I). Analysis of scRNAseq data from adult mice (42) demonstrates that NR3C1 is widely expressed in all pituitary cell types with highest expression in somatotropes and corticotropes (Fig. 1E).

### Characterization of *Prop1^T2AiCre^* mice

**Figure 3:**
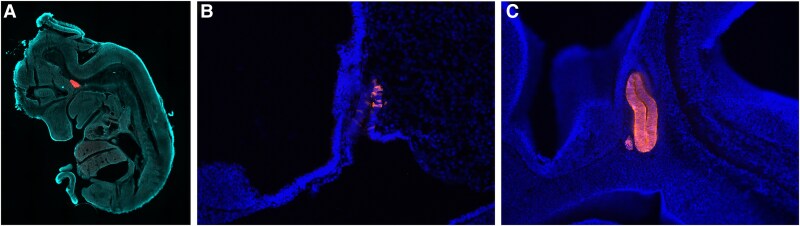
*Prop1^T2AiCre^* mice exhibit efficient cre activity early in pituitary gland development. A, *Stop^fl/fl^-CAG-RFP;Prop1^+/T2AiCre^* embryos show strong red fluorescent protein expression in the Rathke pouch at e12.5 but not in other regions of the embryo. B, *Stop^fl/fl^-CAG-tdTomato;Prop1^+/T2AiCre^* embryos begin to exhibit tdTomato-positive cells by e10.5 in the Rathke pouch. C, Nearly all cells in the Rathke pouch exhibit recombination by e12.5.

To evaluate the role of NR3C1 in the pituitary gland, both developmentally and postnatally, we generated a *Prop1^+/T2AiCre^* mouse line in which floxed DNA is excised efficiently by e12.5 (Fig. 3A). This model improves upon previous *Prop1-cre* lines (48, 49) by eliminating leaky expression of cre seen in transgenic models and by using the T2A system to improve cre expression, increasing the efficiency of DNA excision. To determine the spatial and temporal patterns of cre activity we mated *Prop1^+/T2AiCre^* mice to a reporter mouse with a floxed stop cassette in the ROSA26 locus upstream of a tdTomato reporter (37). We observe cre-responsive recombination initiating by e10.5 in the RP with essentially all cells of the RP expressing the reporter by e12.5 (Fig. 3B and 3C). We also see recombination in granulosa cells, similar to observations in another *Prop1*-*cre* knockin model (49), suggesting that *Prop1* may be briefly expressed in granulosa cells (Supplementary Fig. S1 (50)).

### Number of somatotropes is reduced with loss of *Nr3c1*

**Figure 4:**
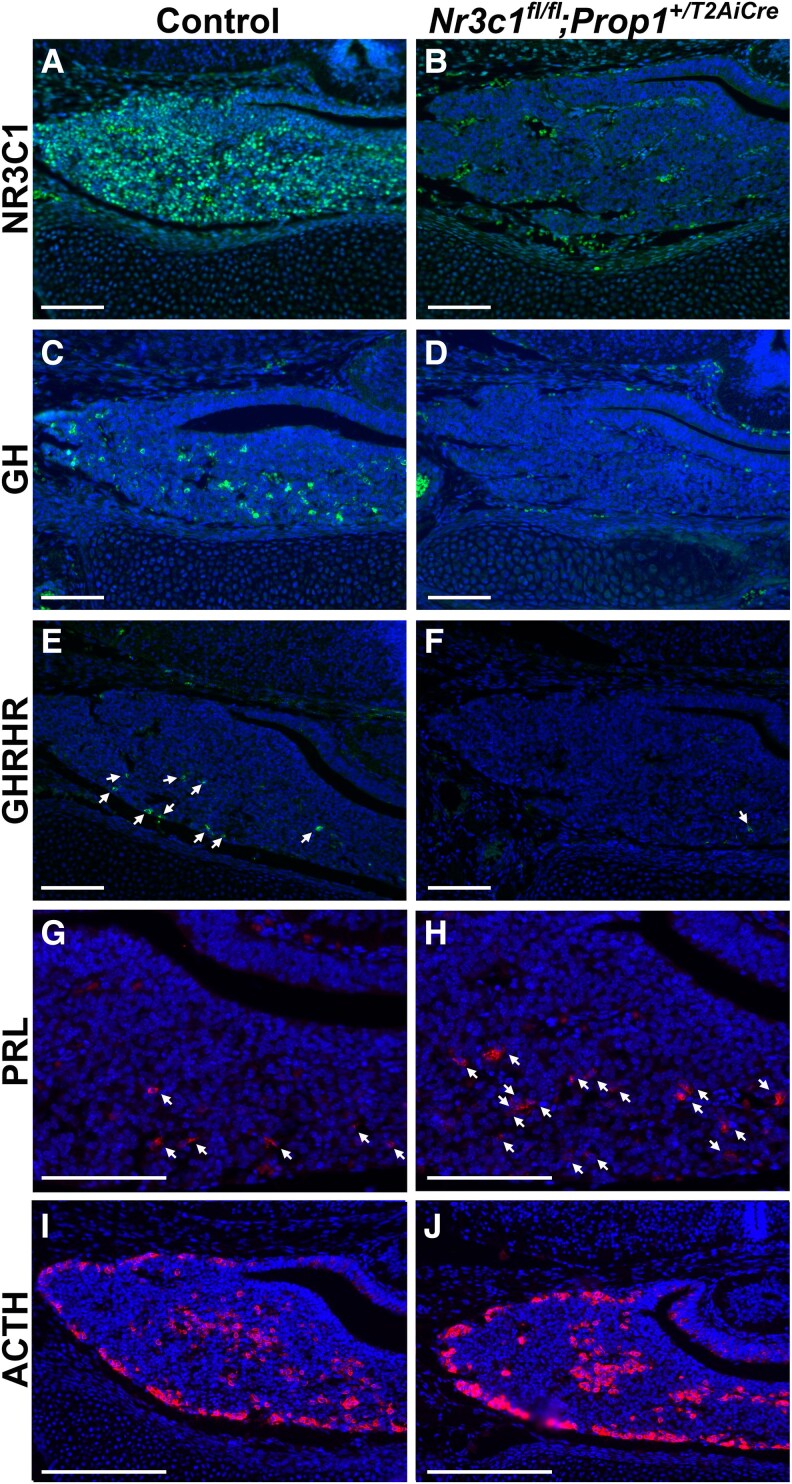
*Nr3c1^fl/fl^;Prop1^+/T2AiCre^* mice exhibit a loss of FOXO1 and impaired somatotrope differentiation at e16.5. Midcoronal sections from *Nr3c1^fl/fl^;Prop1^+/T2AiCre^* embryos and control littermates at e16.5 were analyzed for A and B, NR3C1; C and D, GH; E and F, growth hormone–releasing hormone receptor (GHRHR); G and H, prolactin (PRL), and I and J, adrenocorticotropin (ACTH). Arrows indicate E and F, GHRHR-positive cells, and G and H, PRL-positive cells. Nuclei are stained with DAPI (4′,6-diamidino-2-phenylindole). Images are taken at 20×. Scale bars represent 100 μm. The experiments were replicated independently in 3 different litters.

Glucocorticoid signaling promotes somatotrope differentiation (21, 31, 33, 34, 51). Deletion of *Nr3c1* using the *Cga-cre* results in decreased *Gh1* expression at e18.5 and perinatal lethality (31). To determine the requirement for NR3C1 in somatotrope differentiation, we analyzed mouse embryos lacking NR3C1 at e16.5, the time when somatotropes are first apparent by IHC (20, 30, 52). We evaluated *Nr3c1^fl/fl^;Prop1^+/T2AiCre^* and wild-type (WT) littermates and find that NR3C1 is almost entirely absent at e16.5 (Fig. 4A and 4B), demonstrating efficient excision of *Nr3c1* by *Prop1^+/T2Aicre^* even in development. To evaluate somatotrope differentiation, we performed IHC for GH and GH–releasing hormone receptor (GHRHR). Our results show that while a few GH-positive cells and GHRHR-positive cells are apparent in control embryos, both are almost entirely absent in pituitaries lacking NR3C1 (Fig. 4C-4F).

Glucocorticoids exert a suppressive effect on the differentiation of lactotropes in the fetal rat pituitary, contrasting with their stimulatory influence on GH production (53). In the mouse pituitary gland, lactotropes begin to appear in small numbers at e16.5, but their expression increases significantly after e18.5 and continues to rise postnatally. Additionally, evidence suggests a close developmental relationship between GH and PRL cells, both of which are derived from the same POU1F1-positive precursor cells (10, 11). To explore this relationship further, we examined changes in lactotrope numbers in *Nr3c1^fl/fl^;Prop1^+/T2AiCre^* embryos. Our findings reveal that ablation of NR3C1 leads to an increase in PRL-immunopositive cells compared to controls (Fig. 4G and 4H; Supplementary Fig. S2A (50)). This suggests that NR3C1 plays an important role in regulating the normal differentiation both of lactotropes and somatotropes in the fetal mouse pituitary.

Regulation of the hypothalamic-pituitary-adrenal (HPA) axis depends on negative feedback at the level of the pituitary gland and the hypothalamus (54). Studies in zebrafish show that loss of glucocorticoid signaling leads to an increase in the number of corticotropes during embryonic development due to loss of negative feedback (55). To determine how eliminating glucocorticoid signaling in the pituitary gland affects corticotrope number in the developing mouse, we performed IHC for ACTH on *Nr3c1^fl/fl^;Prop1^+/T2AiCre^* embryos at e16.5. The number of corticotrope cells is not different in *Nr3c1^fl/fl^;Prop1^+/T2AiCre^* mouse embryos compared to WT littermates (Fig. 4I and 4J; Supplementary Fig. S2B (50)). This may be due to the pituitary specific loss of glucocorticoid signaling in our model.

**Figure 5:**
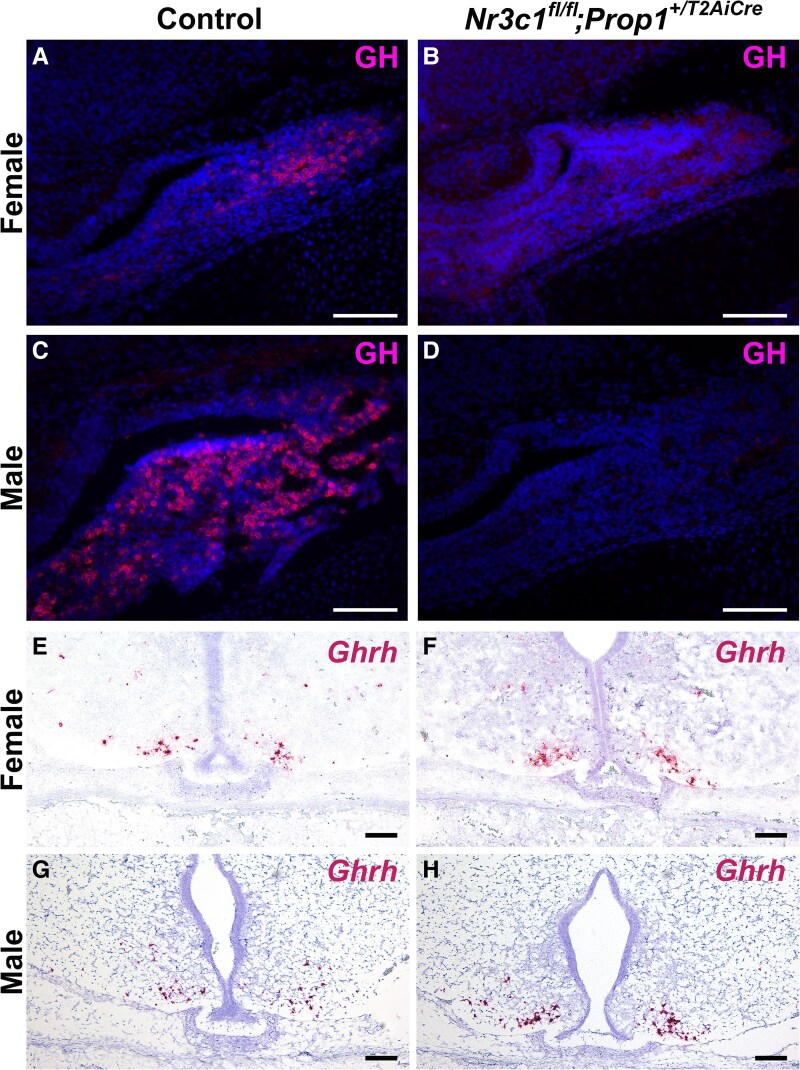
Somatotrope differentiation is severely impaired at e18.5. Midcoronal sections from *Nr3c1^fl/fl^;Prop1^+/T2AiCre^* embryos and control littermates at e18.5 were analyzed for A to D, growth hormone (GH), and E to H, *Ghrh*. Scale bars represent 100 μm. The experiments were replicated independently in 2 different litters for males and females.

To determine if emergence of somatotropes continues to be suppressed later in development, we analyzed somatotrope number at e18.5 in *Nr3c1^fl/fl^;Prop1^+/T2AiCre^* embryos and WT littermates. We found that the number of GH-immunopositive cells is drastically reduced at e18.5 (Fig. 5A-5D), similar to the phenotype of e16.5 pituitary glands. High glucocorticoid levels can suppress hypothalamic *Ghrh* expression (56). To determine if the loss of GH-immunopositive cells could be due to impaired *Ghrh* expression, we performed in situ hybridization for hypothalamic *Ghrh*. *Ghrh* expression was normal in *Nr3c1^fl/fl^;Prop1^+/T2AiCre^* embryos at e18.5 (Fig. 5E-5H). This suggests that loss of glucocorticoid signaling specifically in the pituitary gland does not lead to increased glucocorticoid levels and that the loss of GH in the pituitary gland is not due to a loss of *Ghrh*.

**Figure 6:**
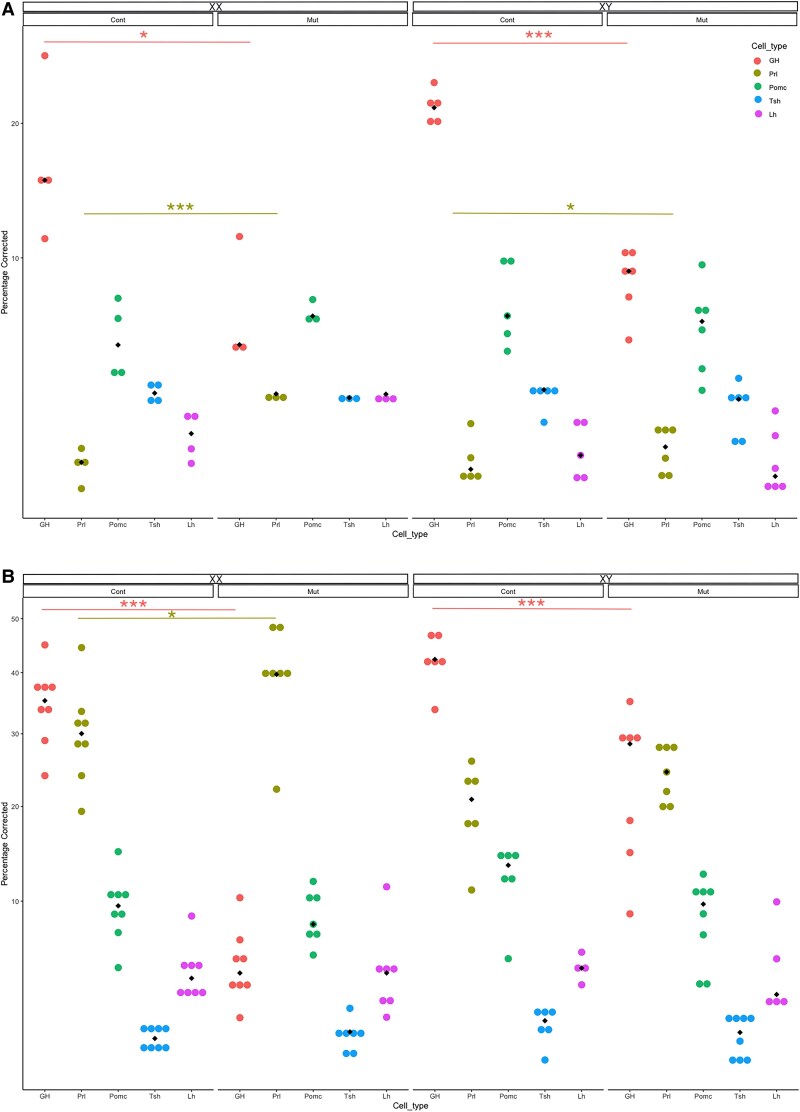
Somatotropes are reduced postnatally in *Nr3c1^fl/fl^;Prop1^+/T2AiCre^* mice. Pituitary cells from A, P5, and B, 7 week old *Nr3c1^fl/fl^;Prop1^+/T2AiCre^* and control mice were dispersed, stained for pituitary hormones by immunohistochemistry, and counted; *P less than .05; ***P less than .001. Y-axis values represent the square root of the percentage of cells counted. Diamonds represent the median for each group.

While *Nr3c1;Cga-cre* mice die at birth (31) possibly due to loss of glucocorticoid signaling in the lung, the *Prop1^T2AiCre^* is more specific, allowing the animals to live into adulthood. To determine if *Nr3c1^fl/fl^;Prop1^+/T2AiCre^* mice continue to exhibit hypopituitarism postnatally, we examined these mice at postnatal day (P)5 and age 7 weeks. While all endocrine cells are represented, the number of somatotropes is reduced in *Nr3c1^fl/fl^;Prop1^+/T2AiCre^* mice at P5 and adult (Fig. 6), consistent with our findings in the embryo. The number of lactotropes is also significantly increased in *Nr3c1^fl/fl^;Prop1^+/T2AiCre^* mice of both sexes at P5 and in adult *Nr3c1^fl/fl^;Prop1^+/T2AiCre^* female but not male mice (see Fig. 6). Similarly, we observe fewer somatotropes in adult males and females (see Fig. 6). This trend is also apparent at P21 (Supplementary Fig. S3 (50)). Interestingly, the somatotropes that are present appear larger with brighter GH immunoreactivity, especially in females (Supplementary Figs. S4 and S5 (50)).

### Loss of NR3C1 alters expression and cellular localization of pituitary transcription factors

**Figure 7:**
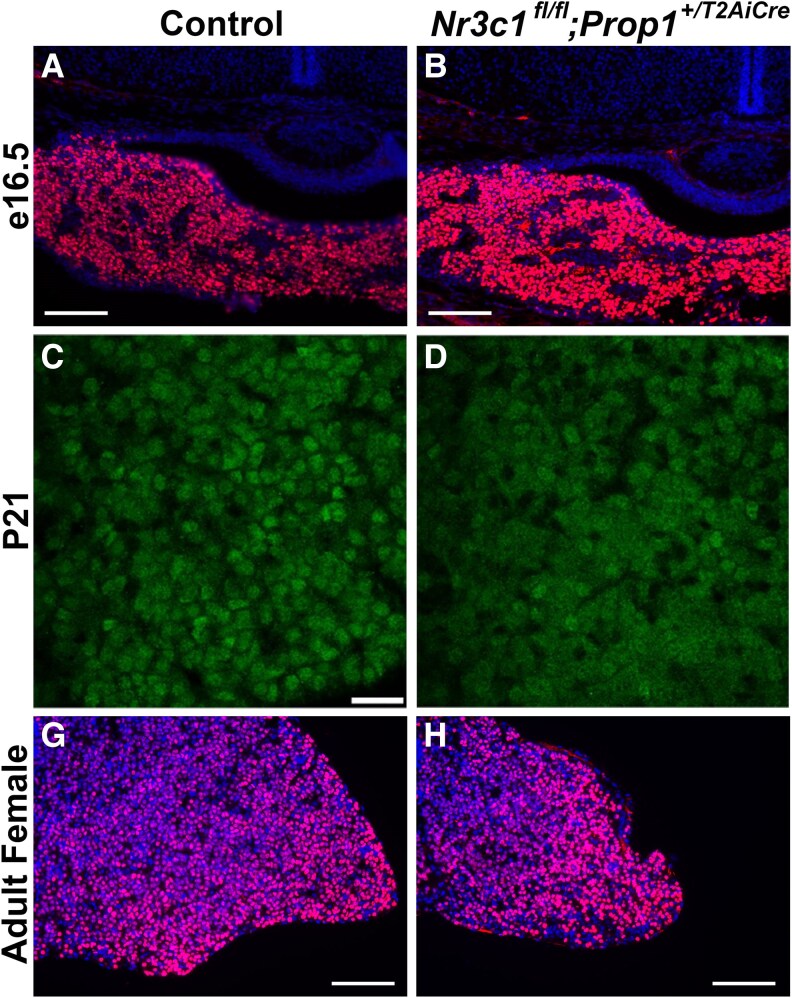
Characterization of POU1F1 in *Nr3c1^fl/fl^;Prop1^+/T2AiCre^* mice. Midcoronal sections from *Nr3c1^fl/fl^;Prop1^+/T2AiCre^* and control mice were analyzed by immunohistochemistry for POU1F1 at A and B, e16.5; C and D, P21; and G and H, age 7 weeks. A and B and G and H, Nuclei are stained with DAPI (4′,6-diamidino-2-phenylindole). Images are taken at 20×. A and B and G and H, Scale bars represent 100 μm. Experiments were replicated in 2 or 3 different animals.

Somatotropes, lactotropes, and thyrotropes derive from a common progenitor that expresses the transcription factor, POU1F1 (10, 11). While we do not observe a change in the number of POU1F1-positive cells, POU1F1 is less nuclear in *Nr3c1^fl/fl^;Prop1^+/T2AiCre^* mice at age 3 weeks (Fig. 7). This suggests that POU1F1 localization may be regulated by glucocorticoid signaling at specific developmental time points, but glucocorticoids do not influence the commitment of progenitor cells to the POU1F1-positive lineage.

**Figure 8:**
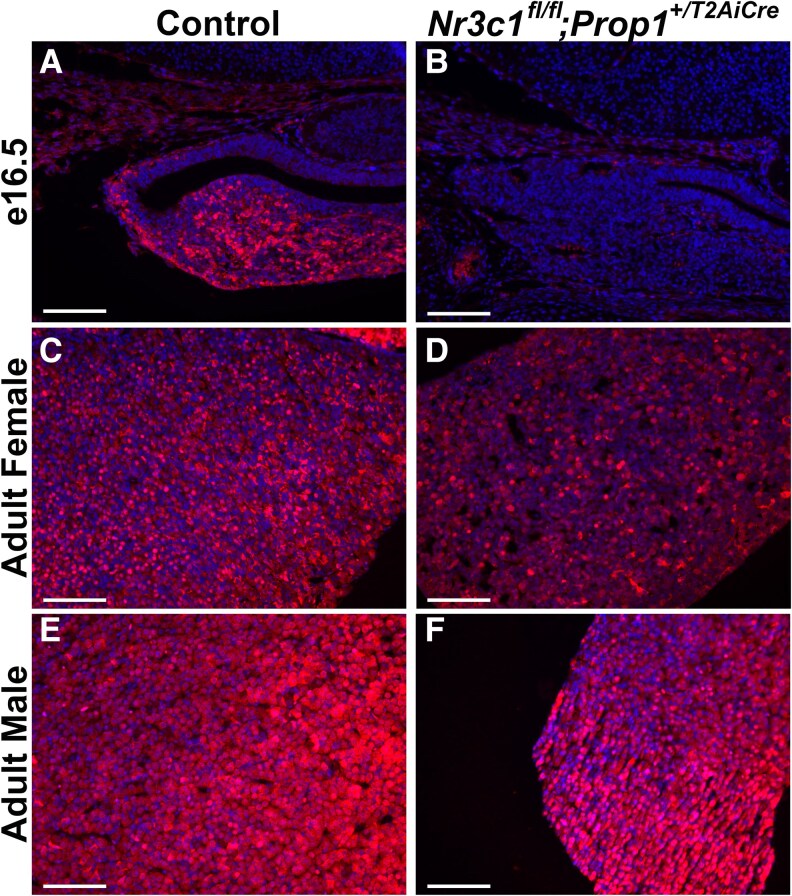
The number of FOXO1-positive cells is reduced in *Nr3c1^fl/fl^;Prop1^+/T2AiCre^* mice. Midcoronal sections from *Nr3c1^fl/fl^;Prop1^+/T2AiCre^* and control mice were analyzed by immunohistochemistry for FOXO1 at A and B, e16.5; C and D, 7-week-old female; and E and F, male mice. Nuclei are stained with DAPI (4′,6-diamidino-2-phenylindole). The experiments were conducted in 3 different litters. Images are taken at 20×. Scale bars represent 100 μm.

Previous studies demonstrate that FOXO1 expression is upregulated by glucocorticoids via glucocorticoid response elements within the *Foxo1* promoter (68). FOXO1 is essential for the differentiation of somatotropes (15), and FOXO1 and GH colocalize with NR3C1 (see Fig. 2). Previously, we demonstrated that glucocorticoids are sufficient to stimulate *Foxo1* expression and nuclear localization (30). We next sought to determine if glucocorticoid signaling is necessary for *Foxo1* expression in pituitary cells. We find that FOXO1 is nearly undetectable in pituitary glands from *Nr3c1^fl/fl^;Prop^+/T2AiCre^* embryos at e16.5 (Fig. 8A and 8B). In adults the number of FOXO1-positive cells is reduced and the proportion of cells containing FOXO1 in the cytoplasm is increased (Fig. 8C and 8D).

## Discussion

Pituitary gland development is a highly dynamic process regulated by a complex interplay of transcription factors and both cell-cell and hormonal signals, each contributing to the differentiation and maturation of specific cell lineages. The somatotrope lineage is significantly influenced by glucocorticoids. A key player in this process is the glucocorticoid receptor, NR3C1 (32, 46). This is particularly interesting, as our results demonstrate a substantial number of NR3C1-positive cells at e16.5 in mouse embryos colocalize with GH-positive cells. NR3C1 and GH exist in the same cells, supporting the hypothesis that NR3C1 is important for somatotrope differentiation and function. These results align with our observations that *Nr3c1^fl/fl^;Prop1^+/T2AiCre^* mice exhibit delayed emergence of somatotrope cells and a reduced number of somatotropes postnatally, strongly suggesting that NR3C1 is a critical regulator of somatotrope emergence embryonically and the wave of somatotrope expansion that occurs postnatally. Consistent with this, we previously found that early activation of the glucocorticoid signaling pathway causes premature somatotrope differentiation (30), suggesting that glucocorticoids are a critical signal governing somatotrope differentiation. Manipulation of other signaling pathways also leads to premature pituitary cell differentiation. For example, disruption of NOTCH signaling through deletion of the NOTCH targets, *Hes1* and *Prop1*, leads to premature differentiation of corticotropes and CGA-producing cells. This suggests that NOTCH signaling is important for preventing pituitary progenitor differentiation (57).

One caveat of this study is the fact that we use GH and GHRHR to identify somatotropes. It is possible that somatotropes have differentiated but are expressing GH and GHRHR at undetectable levels. However, at P5 and in adults the remaining somatotropes are easily detectible, comparable to controls. Therefore, the most likely explanation is rather that the number of somatotropes is reduced, while lactotropes are expanded in females. This is in line with observations of other lineages, such as gonadotropes in the absence of GnRH signaling, where hormone-positive cells are still detected. Contents are affected, but this does not affect detection by immunostaining (58, 59).

Despite the marked reduction in FOXO1 and GH expression in *Nr3c1^fl/fl^;Prop1^+/T2AiCre^* mice, the expression of POU1F1, a key transcription factor for commitment of progenitor cells to the somatotrope, lactotrope, and thyrotrope lineages, remains unchanged at e16.5. This suggests that while POU1F1-positive progenitor cells are committed to becoming either somatotropes, lactotropes or thyrotropes, NR3C1 is necessary for their final maturation into functional, GH-producing cells. This finding builds on previous research that has demonstrated tissue-specific roles for NR3C1 in regulating differentiation across various cell types (60, 61), highlighting its function as a fine-tuner of cellular differentiation rather than a driver of initial lineage commitment. While POU1F1 remains localized to the nucleus in *Nr3c1^fl/fl^;Prop1^+/T2AiCre^* mice at e16.5 and in adult, at P21 POU1F1 is present both in the nucleus and cytoplasm in the *Nr3c1^fl/fl^;Prop1^+/T2AiCre^* mice. This suggests that glucocorticoids may regulate POU1F1 localization at specific developmental stages.

An important question is what signals push POU1F1-positive progenitor cells to differentiate into either somatotropes or lactotropes. We find that a subset of POU1F1-positive cells is also NR3C1 positive. An intriguing possibility is that these cells represent committed presomatotropes. Consistent with this idea, glucocorticoids promote the differentiation of somatotropes (30, 62) and suppress the differentiation of lactotropes (53). For example, glucocorticoids inhibit expression of the bovine *Prl* gene in an OCT1 and PBX-dependent manner (63, 64) and act on the proximal promoter region of the human *PRL* gene to repress its activity, reducing PRL secretion (65-67). Our study expands on this by showing that in *Nr3c1^fl/fl^;Prop1^+/T2AiCre^* mice, lactotrope numbers increase in comparison to controls, suggesting a shift that likely reflects a broader developmental cell fate in the pituitary, where glucocorticoid signaling may normally regulate the balance between somatotrope and lactotrope differentiation from *Pou1f1* progenitors. Similarly, a study by Klug et al (31) demonstrated that deleting *Nr3c1* using the *Cga-cre* led to a decrease in *Gh1* transcripts. In contrast to our findings, no statistically significant change in *Prl* transcript was detected at e17.5 to e18.5. However, there was a strong upward trend in *Prl* expression that may have become significant with more samples. Shifts in cell identity have been described for other pituitary cell types as well. For example, deletion of the NOTCH effector, *Hes1*, causes intermediate lobe progenitor cells to differentiate into somatotropes instead of melanotropes (68).

One of the transcription factors intricately involved in somatotrope differentiation is FOXO1. *Foxo1* deletion phenocopies the impaired somatotrope differentiation seen in *Nr3c1^fl/fl^;Prop1^+/T2AiCre^* embryos at e16.5 (20). However, the somatotrope phenotype at e18.5 is much more severe in *Nr3c1^fl/fl^;Prop1^+/T2AiCre^* embryos, suggesting that FOXO1 is not the only mechanism by which glucocorticoid signaling promotes somatotrope differentiation. FOXO1 is upregulated in response to glucocorticoids in the embryonic pituitary as well as in somatotrope-derived cells (30). The colocalization of NR3C1 and FOXO1 in somatotropes highlights a dynamic regulatory interplay between these two factors, emphasizing NR3C1's pivotal role in maintaining the differentiation program of somatotropes. Interestingly, deletion of *Nr3c1* in mouse pituitary tissue not only results in a loss of FOXO1 but also impaired GH production, reinforcing the idea that glucocorticoid-induced somatotrope differentiation is mediated in part by FOXO1.

GHRH signaling is important for postnatal somatotrope lineage expansion (69) and loss of GHRH signaling leads to a decrease in the number of somatotropes and an increase in the number of lactotropes and *Prl* expression (24). Glucocorticoids strongly stimulate expression of the receptor for GHRH and thus may contribute to this phenomenon (30, 62). Consistent with this, we observe a severe reduction in GHRHR-positive cells in the absence of NR3C1. Thus, the reduction in somatotrope number in *Nr3c1^fl/fl^;Prop1^+/T2AiCre^* mice may be due, in part, to a loss of glucocorticoid-stimulated *Ghrhr* expression. Another important regulatory signal is estradiol. Estradiol promotes lactotrope differentiation and exposure to glucocorticoids reduces lactotrope sensitivity to estradiol (70). We observe a statistically significant decrease in the number of somatotropes and a significant increase in the number of lactotropes both in male and female *Nr3c1^fl/fl^;Prop1^+/T2AiCre^* mice at P5. However, in adults lactotropes are increased in females but not males, suggesting that estradiol may be important for the increase in lactotropes seen in the absence of glucocorticoid signaling.

An important consideration is that *Nr3c1* has been deleted in all pituitary cell types including corticotropes, which could disrupt the HPA axis. Disruption of the HPA axis by adrenalectomy leads to an increase in the number of pituitary corticotropes (71, 72). Global reduction in NR3C1 activity leads to increased glucocorticoid levels in newborn and 6-month-old mice due to reduced negative feedback (73-75). However, deletion of *Nr3c1* in corticotropes using the *Pomc*-*cre* does not increase basal corticosterone levels in 3-month-old mice (76, 77). Deletion of *Nr3c1* in the brain impairs negative feedback and causes elevated glucocorticoid levels in adult mice (78). Consistent with these data, we do not observe a statistically significant difference in corticotrope number in *Nr3c1^fl/fl^;Prop1^+/T2AiCre^* mice at age 7 weeks. This suggests that negative feedback to the hypothalamus, in which NR3C1 is still functional, is sufficient to maintain normal glucocorticoid levels in adults. Interestingly, P6 mice in which *Nr3c1* was deleted in corticotropes using *Pomc-cre* had elevated corticosterone and ACTH levels (77). However, we do not observe a difference in corticotrope number in *Nr3c1^fl/fl^;Prop1^+/T2AiCre^* P5 mice. These findings suggest that although corticotrope number is not increased postnatally, corticotropes may transiently secrete elevated levels of ACTH, which resolves before adulthood.

Fewer studies address the role of negative feedback on corticotropes during embryonic development. An elegant study in zebrafish demonstrates that corticotrope number increases when glucocorticoid synthesis is inhibited and, conversely, corticotropes are reduced in response to administration of exogenous glucocorticoids (55). However, we observe no statistically significant change in the number of corticotropes in *Nr3c1^fl/fl^;Prop1^+/T2AiCre^* mouse embryos. This could reflect species differences or differential effects in global vs pituitary specific disruption of glucocorticoid signaling.

Mutations in *NR3C1* have been associated with Cushing disease (79), which is caused by ACTH-secreting pituitary neuroendocrine tumors. These mutations result in a global reduction in glucocorticoid signaling. In light of studies showing that global loss of glucocorticoid signaling leads to increased corticotrope number (72) and elevated glucocorticoid levels (73, 74), while pituitary specific loss of glucocorticoid signaling does not affect corticotrope number (see Fig. 6) or glucocorticoid levels in adults (76, 77), and disruption of glucocorticoid signaling in the hypothalamus does result in increased levels of ACTH and glucocorticoids, one might hypothesize that ACTH-secreting pituitary neuroendocrine tumors are due to loss of negative feedback to the hypothalamus or the combined loss of negative feedback to the hypothalamus and pituitary gland.

In conclusion, our study demonstrates that NR3C1 plays critical roles at several steps of somatotrope differentiation in the developing mouse pituitary, likely in concert with FOXO1. These findings offer important insights into the molecular mechanisms of pituitary development and may have broader implications for understanding the role of glucocorticoids in somatotrope differentiation and function. Further studies will be needed to elucidate the detailed regulatory pathways involved and to explore the full extent of NR3C1's role in somatotrope differentiation.

## Data Availability

Original data generated and analyzed during this study are included in this published article or in the data repositories listed in “References.”

## Current affiliations

P.D.’s current affiliation is the University of Chicago, Chicago, Illinois, USA. K.R.’s current affiliation is the Centre for Endocrinology, William Harvey Research Institute, Faculty of Medicine and Dentistry, Queen Mary University of London, London, UK.

## Abbreviations

ACTH: adrenocorticotropin
DAPI: 4′,6-diamidino-2 phenylindole
e: embryonic day
GH: growth hormone
GHRH: growth hormone–releasing hormone
GHRHR: growth hormone–releasing hormone receptor
HPA: hypothalamic-pituitary-adrenal
IHC: immunohistochemistry
P: postnatal
PRL: prolactin
qPCR: quantitative polymerase chain reaction
RP: Rathke’s pouch
scRNAseq: single-cell RNA sequencing
TSH: thyrotropin
WT: wild-type
